# Supplementary material for: Partnered Excited-State Intermolecular Proton Transfer Fluorescence (P-ESIPT) Signaling for Nitrate Sensing and High-Resolution Cell-Imaging
Source: Molecules. 2022 Aug 13;27(16):5164. doi: 10.3390/molecules27165164 (PMC9416243; doi:10.3390/molecules27165164)
Supplement: Supplementary file 1 [file molecules-27-05164-s001.zip › molecules-1823211-supplementary.pdf]

molecules

MDPI

Supporting Information for

Partnered excited-state intermolecular proton transfer fluorescence (P-ESIPT) signaling for nitrate sensing and high resolution cell-imaging

Pan Ma, Fuchun Gong,* Hanming Zhu, You Qian, Lingzhi He, Jiaoyun Xia, Zhong Cao

College of Chemistry and Chemical Engineering, Changsha University of Science and Technology, Changsha, 410114, P. R. China

∗Corresponding authors

Tel: +86-0731-85258733;

E-mail addresses: gongfc139@163.com (F.-C. Gong)

Table of contents

1. Characterization of ESIPT-F…………………………….... 2-4

2. Determination of fluorescence quantum yield…………………. 5

3. MTT assays of ESIPT-F………………………………………. 6

1. Characterization of ESIPT-F

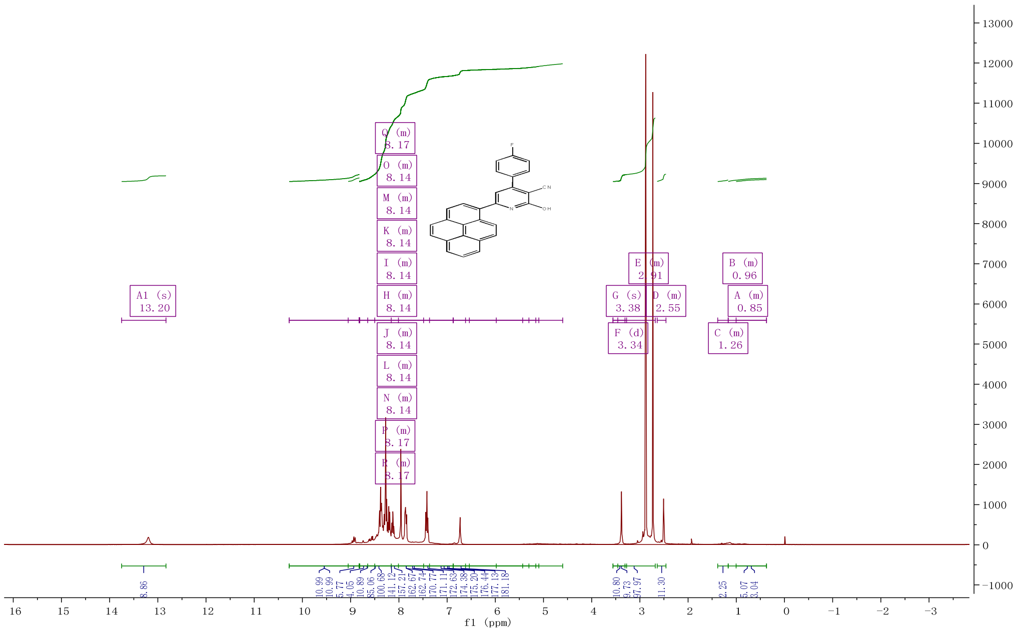

Fig.S1 ^{1}HNMR of ESIPT-F

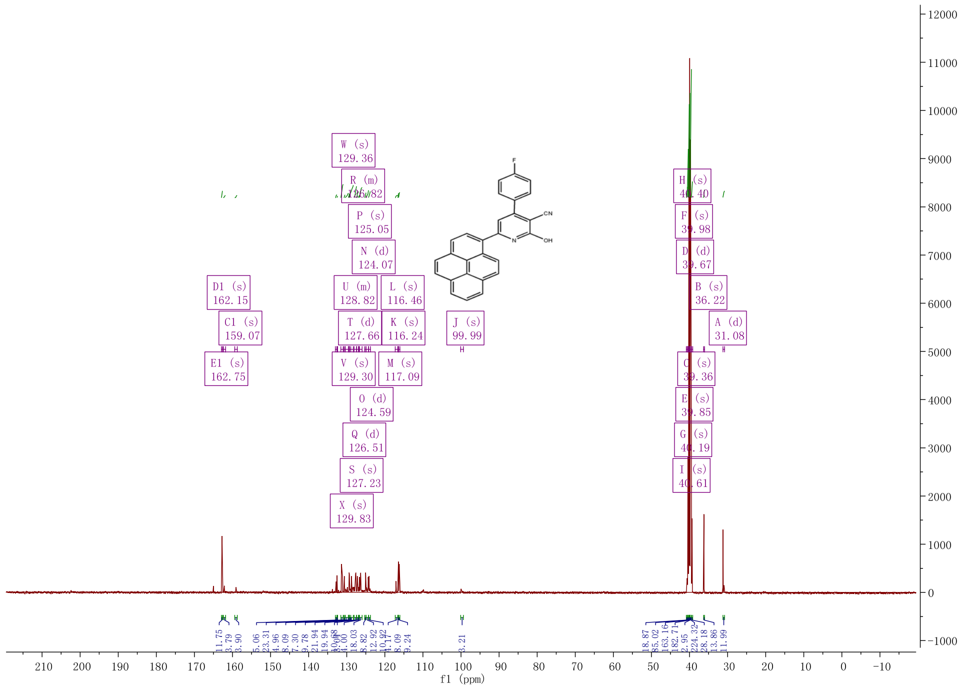

Fig.S2 ^{13}CNMR of ESIPT-F

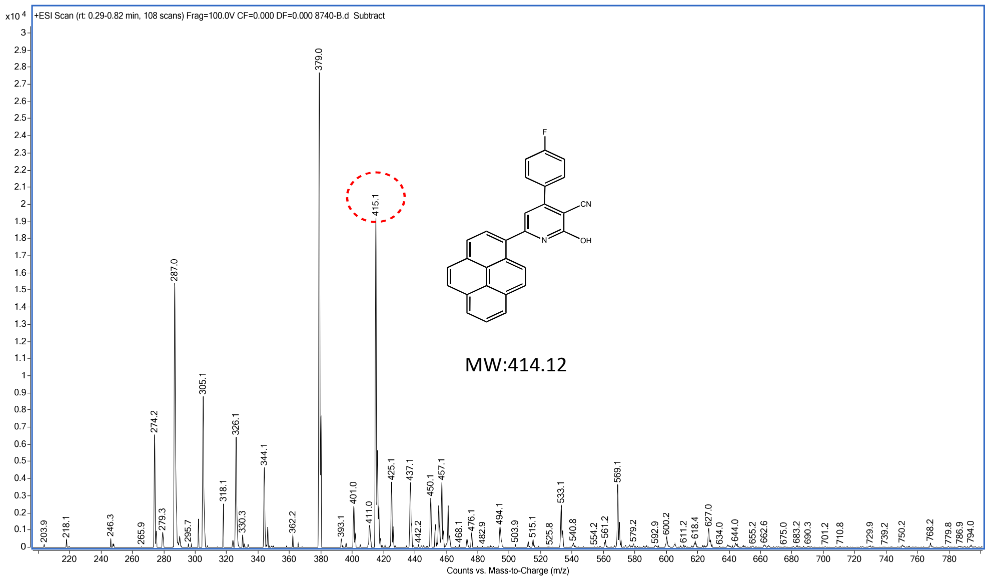
+ESI Scan (rt: 0.29-0.82 min, 108 scans) Frag=100.0V CF=0.000 DF=0.000 8740-B.d Subtract
x10 4
Counts vs. Mass-to-Charge (m/z)
379.0
415.1
287.0
305.1
274.2
326.1
344.1
MW:414.12
F
CN
N
OH

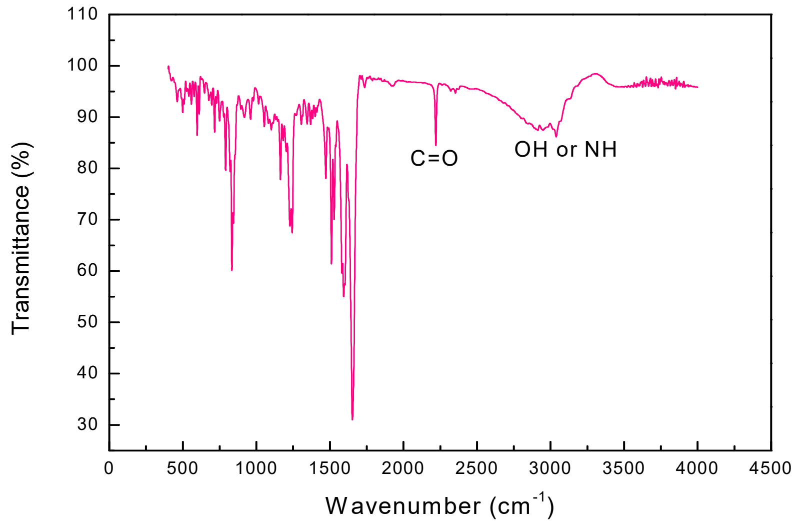

Fig.S4 FT-IR of ESIPT-F

2. Determination of fluorescence quantum yield

The fluorescence quantum yield (FQY) were determined using quinine sulfate (FQY=0.54 at 360 nm) in sulfuric acid (0.10 mol.L^{-1}, η =1.33) as the standard substance. The absolute FQY values were calculated corresponding to the following equation:

$$\Phi_u=\Phi_s(I_u/I_s)(A_s/A_u)(\eta^2{}_u/\eta^2{}_s)$$

Φ is fluorescence quantum yield; I is the measured integrated fluorescence intensity; A is the optical density measured at the selected excitation wavelength; η is the refractive index. The subscript "*s*" refers to the standard FQY of the reference quinine sulfate. The subscript "*u*" refers to the unknown FQY of the fluorescent PNPs. In order to minimize re-absorption effect, absorbance in the 1.0 cm fluorescence cuvette was kept under 0.1 at the excitation wavelength of 360 nm.

3. MTT assays of ESIPT-F

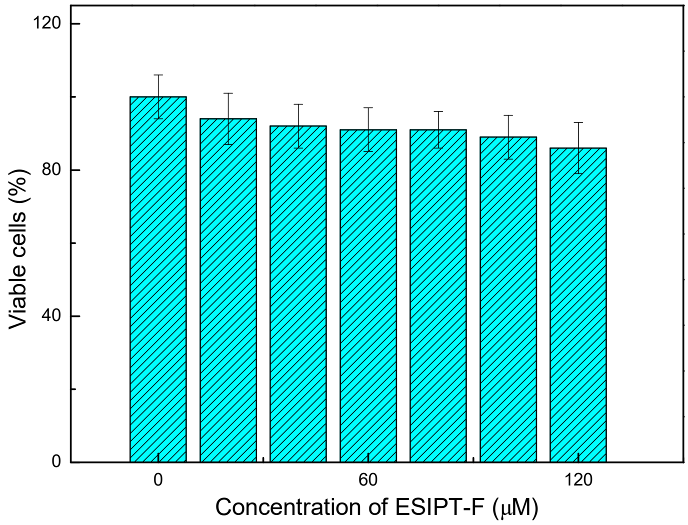

Fig.S5 Denotes% cell viability of HEC-1-A cells treated with different concentrations (0-120 μM) of ESIPT-F for 12 h determined by MTT assay. Results are expressed as mean ± S.D. of three independent experiments.
